# Paramedic-performed Prehospital Point-of-care Ultrasound for Patients with Undifferentiated Dyspnea: A Pilot Study

**DOI:** 10.5811/westjem.2020.12.49254

**Published:** 2021-03-24

**Authors:** Jacob H. Schoeneck, Ryan F. Coughlin, Cristiana Baloescu, David C. Cone, Rachel B. Liu, Sharmin Kalam, Amanda K. Medoro, Ian Medoro, Daniel Joseph, Kevin Burns, Jesse I. Bohrer-Clancy, Christopher L. Moore

**Affiliations:** * Yale University School of Medicine, Department of Emergency Medicine, New Haven, Connecticut; † Wake Forest University School of Medicine, Department of Emergency Medicine, Winston-Salem, North Carolina

## Abstract

**Introduction:**

Thoracic ultrasound is frequently used in the emergency department (ED) to determine the etiology of dyspnea, yet its use is not widespread in the prehospital setting. We sought to investigate the feasibility and diagnostic performance of paramedic acquisition and assessment of thoracic ultrasound images in the prehospital environment, specifically for the detection of B-lines in congestive heart failure (CHF).

**Methods:**

This was a prospective observational study of a convenience sample of adult patients with a chief complaint of dyspnea. Paramedics participated in a didactic and hands-on session instructing them how to use a portable ultrasound device. Paramedics assessed patients for the presence of B-lines. Sensitivity and specificity for the presence of bilateral B-lines and any B-lines were calculated based on discharge diagnosis. Clips archived to the ultrasound units were reviewed and paramedic interpretations were compared to expert sonologist interpretations.

**Results:**

A total of 63 paramedics completed both didactic and hands-on training, and 22 performed ultrasounds in the field. There were 65 patients with B-line findings recorded and a discharge diagnosis for analysis. The presence of bilateral B-lines for diagnosis of CHF yielded a sensitivity of 80.0% (95% confidence interval [CI], 51.4–94.7%) and specificity of 72.0% (95% CI, 57.3–83.3), while presence of any B-lines was 93.3% sensitive (95% CI, 66.0–99.7%), and 50% specific (95% CI, 35.7–64.2%) for CHF. Paramedics archived 117 ultrasound clips of which 63% were determined to be adequate for interpretation. Comparison of paramedic and expert sonologist interpretation of images showed good inter-rater agreement for detection of any B-lines (k = 0.60; 95% CI, 0.36–0.84).

**Conclusion:**

This observational pilot study suggests that prehospital lung ultrasound for B-lines may aid in identifying or excluding CHF as a cause of dyspnea. The presence of bilateral B-lines as determined by paramedics is reasonably sensitive and specific for the diagnosis of CHF and pulmonary edema, while the absence of B lines is likely to exclude significant decompensated heart failure. The study was limited by being a convenience sample and highlighted some of the difficulties related to prehospital research. Larger funded trials will be needed to provide more definitive data.

## INTRODUCTION

Shortness of breath is responsible for more than 10% of non-traumatic emergency medical service (EMS) transports.^1^ Morbidity is high, with half of these patients ultimately admitted to the hospital, and a third of those requiring treatment in intensive care units.^1^ Distinction between types of acute pulmonary pathology has important implications for acute treatment, particularly when congestive heart failure (CHF) can be more definitively identified. The American College of Cardiology Foundation and the American Heart Association recommend early treatment of fluid overload, and early treatment with diuretics has been associated with lower rates of in-hospital mortality.^2^,^3^

Prehospital medications have been associated with improved survival in patients with decompensated CHF, while increased mortality is associated with misdiagnosis.^4^ Thoracic ultrasound is frequently used in the emergency department (ED) to determine the etiology of dyspnea, yet its use is not widespread in the prehospital setting. Prospective studies of prehospital cardiothoracic ultrasound are limited and have tended to focus on determination of cardiac activity and lung sliding by physician operators.^5^–^9^

In this study we sought to investigate the feasibility of training paramedics in acquisition and assessment of thoracic ultrasound images in the prehospital environment. The primary aim was to determine whether assessment of B-lines by paramedics in the prehospital setting could identify patients ultimately diagnosed with pulmonary edema from decompensated heart failure. We also sought to determine accuracy of B-line interpretation based on image capture and/or expert determination of B-lines when this was available.

## METHODS

This was a prospective observational study of a convenience sample of patients being transported by emergency medical services (EMS) with a chief complaint of dyspnea. Patients were enrolled when a trained paramedic was working and had access to one of the shared portable ultrasound units. Paramedics were instructed to enroll as many eligible patients as possible.

Participating paramedics were employed by American Medical Response (AMR) or local fire department-based EMS services and transported patients to two EDs located in New Haven, CT. Each EMS agency or fire department was given two or three portable ultrasound units. Enrolled paramedics participated in a 90-minute didactic training session, which included instruction in the use of a portable, handheld ultrasound device, the Vscan with Dual Probe (GE Healthcare, Chicago, IL), and basic principles of ultrasound, techniques to obtain the necessary thoracic views, as well as review of normal and pathologic video clips. They completed an identical, nine-question multiple-choice pre- and post-test during the didactic session.

**Population Health Research Capsule**

What do we already know about this issue?

*Thoracic ultrasound is frequently used in the emergency department (ED) to determine the etiology of dyspnea, yet its use is not widespread in the prehospital setting.*

What was the research question?

*Can paramedics use ultrasound to identify B-lines in patients ultimately diagnosed with decompensated heart failure?*

What was the major finding of the study?

*The presence of bilateral B-lines determined by paramedics is reasonably sensitive and specific for the diagnosis of pulmonary edema.*

How does this improve population health?

*The early diagnosis of pulmonary edema from decompensated heart failure should allow for more rapid initiation of pathology specific treatments.*

Paramedics then completed a 2–3 hour supervised hands-on session using the portable ultrasound device in the ED, imaging patients presenting with shortness of breath. The goal of this training session was to perform and interpret normal and abnormal scans on patients similar to those they could encounter in the field. During the hands-on sessions, paramedics were expected to correctly identify positive or negative findings in at least six patients presenting with undifferentiated pathology, based on previous competency studies with novice sonographers.^10^ Didactic and hands-on sessions were facilitated and directly supervised by members of the investigatory group, including fellowship-trained ultrasound and EMS attending emergency physicians, ultrasound and EMS fellows, and senior emergency medicine residents.

Patients were eligible for inclusion in the study if they were aged >18 with the chief complaint of shortness of breath, and at least one of the following signs or symptoms of respiratory distress:

1. Respiratory rate > 20 per minute
2. Oxygen saturation < 92%
3. Rales, rhonchi, or wheezing on pulmonary auscultation
4. Increased work of breathing: accessory muscle use, tripoding, nasal flaring.
5. Reported progression of pedal edema or orthopnea.

Permission to perform the ultrasound in the field was obtained by the paramedics using a scripted, brief verbal consent, with full consent obtained by one of the study investigators via telephone during the patient-medic interaction. This study was approved by the investigational review board of Yale University and the Yale-New Haven Hospital Center. Patients were enrolled over a 20-month period at the discretion of the participating paramedics in a convenience sample based on ultrasound device availability and patient volume.

All prehospital ultrasounds were performed by paramedics using a GE Vscan with Dual Probe. In some cases, EMS physicians were present during EMS transport; however, they were specifically instructed not to influence or aid in ultrasound acquisition and interpretation. Paramedics assessed for B-lines with the phased array probe in the second or third intercostal space in the midclavicular line of the right followed by the left anterior chest. Paramedics were provided with standardized data sheets where they noted the presence of any B-lines (1–3) or significant B-lines (>3 in one intercostal space). They were specifically instructed not to alter patient care based on their ultrasound findings, nor to delay standard care to perform the ultrasound. Paramedics were asked to record a clip from each ultrasound view on the device’s removable disk and note the time and date of the ultrasound on the data sheet, so that these images could be matched to the patient for review.

At the conclusion of the study patient charts were reviewed for discharge diagnosis by a single investigator (JS) who was blinded to paramedic ultrasound findings and categorized the discharge diagnosis into CHF/pulmonary edema, chronic obstructive pulmonary disease/asthma, pneumonia, or other. Available images were collected from the ultrasound devices and matched to patient data sheets based on the image timestamps and the times recorded by the paramedic. These video clips were reviewed by an emergency physician (CB) with ultrasound fellowship training (defined as the “expert sonologist”) for adequacy of image acquisition and interpretation. Clips that did not definitively visualize the pleural line were deemed inadequate.

We calculated sensitivity and specificity for the presence of bilateral B-lines and any B-lines with 95% confidence intervals (CI) based on discharge diagnosis. Paramedic interpretations were compared to expert sonologist interpretations using an unweighted Cohen kappa statistic.

## RESULTS

A total of 71 paramedics were enrolled and completed the didactic training. Of these participants, 60 reported no prior ultrasound experience and 11 reported prior ultrasound experience with an average of two estimated total hours using ultrasound before attending the training session. The average pretest score was 76.9%, and the average post-test score was 95.8%. After the didactic training, 63 paramedics completed hands-on training and 22 paramedics performed study ultrasounds in the field.

Initially 69 patients were enrolled in the study; three were excluded due to insufficient identifying information. Paramedics recorded their assessments for the presence or absence B-lines in 65 patients. Patient demographics and clinical characteristics are summarized in Table 1.

Pulmonary edema or CHF was diagnosed in 15 of 65 subjects (23.1%), with any B-lines present in 14 and bilateral B-lines present in 12 subjects (Table 2). The presence of bilateral B-lines for diagnosis of CHF yielded a sensitivity of 80.0% (95% CI, 51.4–94.7%) and specificity of 72.0% (95% CI, 57.3–83.3), while presence of any B-lines was 93.3% sensitive (95% CI, .0–99.7%) and 50% specific (95% CI, 35.7–64.2%) for CHF. The positive predictive value of bilateral B-lines for the diagnosis of CHF was 46.1% (95% CI, 41.0–51.3%), while the presence of any B-lines yielded a positive predictive value of 35.9% (95% CI, 32.4–39.5%).

Paramedics recorded 117 clips from 33 patients on the hand-held ultrasound units; of those images, 63% were adequate for interpretation. Comparison of paramedic and expert sonologist interpretations of archived images showed good inter-rater agreement for detection of any B-lines (k = 0.60, 95% CI, 0.36–0.84).

## DISCUSSION

This is the first study we are aware of to assess the feasibility and utility of training paramedics in prehospital ultrasound for the diagnosis of CHF in the United States EMS system and the first to use an ultra-mobile device. These results show that the presence of bilateral B-lines as determined by EMS is reasonably sensitive and specific for the diagnosis of CHF, while the absence of any B-lines makes the diagnosis unlikely. A previous study in Denmark examining assessment for B-lines in CHF using a larger laptop-sized device (SonoSite Edge) enrolled 40 patients and found a sensitivity of 94% for B-lines in the diagnosis of CHF, concluding that ultrasound could be helpful in excluding CHF, which is consistent with these results.^11^

Another study of prehospital, thoracic ultrasound in medical patients with respiratory distress found that paramedic-performed pulmonary ultrasound with remote physician interpretation did not meet the authors’ predefined standards for feasibility.^12^ The authors noted that failed transmission of images contributed to non-feasibility. Interpretation of point-of-care ultrasound studies by paramedics would eliminate the need for image transfer and will likely be a necessary step in making prehospital ultrasound use feasible. In this study, there was good inter-rater agreement with the expert sonologist review for the presence of any B-lines, although notably this assessment was limited by poor compliance with image archival for review.

While we believe that this study adds to evidence that motivated prehospital providers can be trained to perform and obtain useful information that can potentially impact treatment and patient outcome, conclusions regarding feasibility need to be tempered by the inconsistent use of ultrasound in this setting. While 63 paramedics completed training, only 22 ultimately enrolled subjects, and our overall enrollment was lower than expected given the duration of the study. Further study is needed to identify the barriers to ultrasound performance in this setting and to understand how EMS providers may be more incentivized to incorporate ultrasound into their practice.

The implementation of ultrasound into an EMS system requires a significant initial monetary investment, instructor and paramedic time dedicated to training, and ongoing oversight of user performance for patient safety. Additional study is necessary to determine whether paramedic detection of thoracic pathology can lead to meaningful changes in management that would justify these costs. It seems probable that earlier diagnosis and initiation of pathology-specific treatments would positively impact patient care, although this was outside the scope of our study. Many of these challenges and costs of implementing ultrasound usage are likely to decrease in the future as portable units become more affordable and widespread ultrasound proficiency among graduating emergency physicians increases the availability of instructors.

## LIMITATIONS

This study was limited by relatively low paramedic participation, low enrollment of eligible patients, logistic challenges of retrieving and sharing units, and difficulty recording data and ultrasound images. Prehospital patient care research is consistently challenging for clinicians, and this study was no exception.^13^–^14^ Generating a steady enrollment of patients and maintaining paramedic interest was difficult. Despite 63 paramedics having completed the didactic and hands-on training, only 22 went on to perform documented ultrasounds in the field, with 42% of the 22 paramedics performing only one ultrasound each. Most patients were enrolled in the first month following the paramedic training, with only seven paramedics participating in the study after that point. This inconsistency may have introduced several biases to the data, including that ultrasound was likely performed by paramedics who were more motivated (and thus perhaps more skilled than average), and that ultrasound may have been performed when there was more time and the situation was less acute (ie, less-ill patients). While recent graduates of emergency medicine residencies are proficient in pulmonary ultrasound and likely capable of teaching these skills to paramedics, the results of this study may not be generalizable to smaller EMS systems without the support of ultrasound fellowship-trained physicians.

We are unable to confidently suggest that paramedics retained thoracic ultrasound skills following their training or routinely used them in their patient evaluations. In discussions with participating EMS providers, most felt that ultrasound was useful but noted that difficulty adhering to study protocols, data collection requiring manual input, and limited ultrasound device availability were significant challenges. User interfaces that facilitate input of patient information, documentation of findings, image archival, and submission for review would almost certainly increase the quantity and quality of data collected in futures studies. Additional research focusing on paramedics’ attitudes regarding the utility of ultrasound would also be advantageous in determining how to implement sustained use in prehospital practice patterns.

Average transport times for most patients in our system are less than 10 minutes, rendering ultrasound study completion and full documentation of findings difficult. These short transport times might have also biased paramedics to enroll less-ill patients requiring fewer interventions affording the paramedics the time necessary to perform the ultrasound. Ultrasound may have greater utility in prehospital systems with longer transport times, where paramedics are engaged in prolonged patient management, and misdiagnosis leading to inappropriate treatments is more detrimental. As enrollment was determined by the paramedics, it is possible that there was bias toward selecting patients with a clear diagnosis of CHF or pulmonary edema. In this study, 23% of patients were discharged with a diagnosis of CHF, which is slightly higher than the rate of 16% previously cited in studies of patients presenting to EMS with shortness of breath.^1^

Of the clips archived, 37% were inadequate for interpretation. This suggests that obtaining adequate images in the prehospital setting may be difficult for novice, non-physician sonographers. This may be partially ascribed to the paramedics’ training sessions, which focused primarily on scanning technique, normal findings, and pathology, with less emphasis on the adequacy of images and the importance of archiving for review. On several occasions inadequate images were submitted alongside adequate images for the same patient; it is possible that some of the inadequate images were recorded inadvertently by the paramedics. Ideally, ultrasound units used in the prehospital setting would allow for wireless transmission of images to an archiving system with the capability to include ultrasound interpretations for review. Compliance with image archival protocol would also be improved by user interface restrictions that discourage ultrasound use without entering patient identifiers or saving clips.

Among the logistical challenges of prehospital ultrasound are device fragility, need for charging, and the requirement for physical or wireless connectivity. Advances in portable ultrasound units since the start of this study (including the second-generation Vscan Extend) have already resulted in devices that are more amenable to prehospital use. Many of these devices allow for remote video guidance in scanning technique and image acquisition in real time by a more experienced clinician who is not at the bedside. The advent of capacitive micromachined ultrasonic transducer-based probes may also alleviate some of these issues. Production of these probes is cheaper than their piezoelectric counterparts, making them more easily obtainable for research purposes and affordable for EMS agencies.

## CONCLUSION

This observational pilot study suggests that prehospital lung ultrasound for B-lines may aid in identifying or excluding CHF as a cause of dyspnea. The presence of bilateral B-lines as determined by EMS is reasonably sensitive and specific for the diagnosis of CHF and pulmonary edema, while the absence of B-lines is likely to exclude significant decompensated heart failure. The study was limited by being a convenience sample and highlighted some of the difficulties related to prehospital research. Larger funded trials will be needed to provide more definitive data.

## Figures and Tables

**Table 1:** Demographics and clinical characteristics of patients evaluated by paramedics using a portable device to perform lung ultrasound.

Characteristic	Value
Age	
Average	64 +/− 17 years
Range	19–94 years
Gender	
Male	37 (57%)
Female	28 (43%)
Prehospital vital signs	
Heart rate	93 +/− 21
Respiratory rate	23 +/− 6
Room air oxygen saturation	92 +/− 5%
Oxygen device	
Room air	33 (49%)
Nasal cannula	13 (19.5%)
Non-rebreather mask	13 (19.5%)
CPAP	4 (6%)
No oxygen device recorded	4 (6%)
Discharge diagnosis	
COPD or asthma	21 (32.3%)
Congestive heart failure or Pulmonary edema	15 (23.1)
Pneumonia	5 (7.7%)
Other	24 (36.9%)

*CPAP*, continuous positive airway pressure; *COPD*, chronic obstructive pulmonary disease.

**Table 2:** Paramedic interpretation of B-lines compared with discharge diagnosis.

Discharge diagnosis	Any B-lines bilateral	Any B-lines present	No B-lines present
CHF or pulmonary edema (n = 15)	12 (80%)	14 (93.3%)	1 (6.7%)
COPD or asthma (n = 21)	5 (23.8)	10 (47.6%)	11 (52.4%)
Pneumonia (n = 5)	3 (60%)	5 (100%)	0 (0%)
Other (n= 24)	6 (25%)	10 (41.6%)	14 (58.3%)

*CHF*, congestive heart failure; *COPD*, chronic obstructive pulmonary disease.
